# Monoamine Oxidase A Promoter Variable Number of Tandem Repeats (*MAOA-uVNTR*) in Alcoholics According to Lesch Typology 

**DOI:** 10.3390/ijerph120303317

**Published:** 2015-03-19

**Authors:** Agnieszka Samochowiec, Magdalena Chęć, Edyta Kopaczewska, Jerzy Samochowiec, Otto Lesch, Elżbieta Grochans, Andrzej Jasiewicz, Przemyslaw Bienkowski, Kołodziej Łukasz, Anna Grzywacz

**Affiliations:** 1 Institute of Psychology, Department of Clinical Psychology, University of Szczecin, Krakowska 69, 71-017 Szczecin, Poland; E-Mails: samoaga@tlen.pl (A.S.); magda.chec@gmail.com (M.C.); 2 University Center for Education, University of Szczecin, Szwoleżerów 18a, 71-062 Szczecin, Poland; E-Mail: edi55@wp.pl; 3 Department of Psychiatry, Pomeranian Medical University, Broniewskiego 26, 71-460 Szczecin, Poland; E-Mails: samoj@pum.edu.pl (J.S.); andrzej.jasiewicz@gmail.com (A.J.); 4 Department of Psychiatry, Vienna University, Waehringer Guertel 18–20, A-1090 Vienna, Austria; E-Mail: otto-michael.lesch@meduniwien.ac.at; 5 Department of Nursing, Pomeranian Medical University, Żołnierska 48, 71-210 Szczecin, Poland; E-Mail: grochans@pum.edu.pl; 6 Institute of Psychiatry and Neurology, Department of Pharmacology, Sobieskiego 9, 02-957 Warszawa, Poland; E-Mail: bienkow@ipin.edu.pl; 7 Department of Orthopedics, Pomeranian Medical University, Unii Lubelskiej 1, 71-460 Szczecin, Poland; E-Mail: Lukas@hot.pl

**Keywords:** alcohol dependence, MAOA, Lesch’s typology

## Abstract

*Background:* The aim of this study was to examine the association between the *MAOA-uVNTR* gene polymorphism in a homogeneous subgroups of patients with alcohol dependence categorized according to Lesch’s typology. *Methods:* DNA was provided from alcohol dependent (AD) patients (n = 370) and healthy control subjects (n = 168) all of Polish descent. The history of alcoholism was obtained using the Polish version of the Semi-Structured Assessment for the Genetics of Alcoholism (SSAGA). Samples were genotyped using PCR methods. *Results:* We found no association between alcohol dependence and *MAOA* gene polymorphism. *Conclusions:* Lesch typology is a clinical consequence of the disease and its phenotypic description is too complex for a simple genetic analysis.

## 1. Introduction

Alcohol dependence (AD) is a complex disorder often comorbid with other psychiatric disorders [1,2]. Data provided by family, twin and adoption studies suggest that there is a genetic risk factor for AD and heredity plays an important role in alcohol dependence and drinking behavior [1,3,4]. Alcohol dependence is a multigenic disorder [5]. Several candidate genes have been studied, but results are controversial [6,7,8,9,10,11,12,13,14,15,16,17]. In our last study alcoholics and nonalcoholics did not differ significantly in terms of 3-SNP GABRG1 haplotype frequencies. No significant differences in GABRG1 haplotype distributions were also observed between subgroups of alcoholics selected based on Lesch typology and non-drinking controls [16].

Depending on the family history of alcohol dependence, previous personal psychopathology, and hypothetical neurobiological background [18], four evolutionary types of alcohol dependent subjects were established. According to Lesch typology (LT), type I ADS (the so-called model of “allergy”) individuals suffer from heavy alcohol withdrawal syndrome, probably associated with dopamine deficits, and tend to use alcohol to weaken withdrawal symptoms. Patients of type II (model of anxiety of conflict) use alcohol as self-medication because of its anxiolytic effect. In AD of type III, the main characteristic is of an affective disorder and thus alcohol is used as an antidepressant by these subjects. Type IV patients (alcohol drinking as adaptation) show premorbid cerebral defects, behavioral disorders, and a high social burden [18].

The MAOA enzyme metabolizes monoamine neurotransmitters, including serotonin [19]. The promoter region of MAOA located on the short arm of the X chromosome contains 30 base pair variable number of tandem repeats sequence (VNTR) with 2, 3, 3.5, 4, or 5 repeated copies [19,20]. Transcription of the 3-repeat (short) allele results in reduced MAOA activity and consequently the level of serotonin in the synapse is increased, which, allegedly, increases the risk for aggression and ASB. The frequency of the ‘‘risk’’ allele in non-clinical samples of European ancestry ranges from 0.3 to 0.4, although the frequency of this allele in individuals of Asian and African ancestry seems to be considerably higher [19]. In contrast, the 4-repeat (long) allele results in increased MAOA activity and is regarded as the low-risk allele [20]. Of the less common alleles, the 3.5-repeat has shown evidence of activity similar to that of the 4-repeat and is thus considered high activity, whereas the 2-repeat is normally grouped with the 3-repeat allele and considered low activity [20]. There have been inconsistencies across studies in classification of the 5-repeat allele. A complication arises for MAOA’s location on the X chromosome. Since females have two X chromosomes and males have only one, heterozygosity may be present in females but not males. As MAOA expression for heterozygous allele carriers is still unclear, many investigators have selected all-male samples or eliminated heterozygous females from their samples [21,22,23]. Taking into account the clinical aspect of Lesch typology one polymorphism has been selected for genotyping.

## 2. Materials and Methods

This study included a group of 370 Caucasian subjects (mean age: 44.8 years; 370 males), with no history of psychiatric disorders other than alcohol or nicotine dependence as classified by ICD-10. According to Lesch typology, 105 AD subjects were of type I (mean age: 44.5 years; 105 males), 86 patients of type II (mean age: 45.3 years; 86 males), 92 patients of type III (mean age: 43.5 years; 92 males), and 87 patients of type IV(mean age: 46.4 years; 87 males). The control group (mean age: 38.1 years; 168 males) comprised of 168 unrelated individuals matched for ethnicity, and excluded for mental disorders using the Primary Care Evaluation of Mental Disorders (Prime MD) questionnaire. Recruitment and study of each patient were carried out by the authorized personnel of the Department of Psychiatry, Pomeranian Medical University. The study protocol was approved by the Ethical Committee of Pomeranian Medical University of Szczecin (KB-0012/103/11). All participants gave written informed consent. 

The AD patients were classified by the Lesch typology, using a computerized decision tree [18]. The interview was performed in Polish. According to the LAT interview, patients exhibiting symptoms of severe negative impact on childhood development before the age of 14 such as prenatal trauma, cerebral trauma, CNS diseases, bedwetting after the age of 3, stuttering/nail biting, or seizures even outside withdrawal phase were identified as “organic, Type IV”. Patients not exhibiting these symptoms (“non-organic”) were tested for affective features. If found positive for affective symptoms they were identified as Type III (“affective”), the rest were considered to be part of the Type I or II groups “non-organic non-affective”, a further subdivision between Lesch Type I and II is possible in alcohol addiction. Genomic DNA was extracted from venous blood samples using a salting out method.

### 2.1. Genotyping for MAOA-u 30 bp VNTR

The *MAOA-uVNTR* polymorphism was examined using the PCR method. The following primers were used F: 5’CCC-AGG-CTG-CTC-CAG-AAA-3’, R: 5’-GGA-CCT-GGG-CAG-TTG-TGC-3’. The amplification of DNA fragments was performed in a PTC-200 (MJ Research, St. Bruno (Quebec) Canada) thermal cycler. A 15 µL amplification mixture contained 250 ng of genomic DNA, 0.45 µM of each primer, 0.17 mM of each dNTP, 1.5 mM MgCl_2_, 75 mM Tris-HCl, 20 mM (NH_4_)_2_SO_4_, 0.01% Tween, 0.15% DMSO, and 0.5 U of Taq DNA polymerase (MBI Fermentas, St.Leon-Rot, Germany). The cycling conditions were: initial denaturation 95 °C for 3 min followed by 35 cycles with a profile of 94 °C for 40 s, annealing temperature 57 °C for 35 s and 72 °C for 50 s, final elongation in 72 °C for 7 min. Amplification products were separated by electrophoresis on Metaphor agarose gel. The repeats were visualized by ethidium bromide staining. 30 bp VNTR polymorphism had three alleles: 209 bp 3 reps, 4-repeat: 239 bp and 269 bp 5 reps.

### 2.2. Statistical Analysis

Allele/genotype frequencies in our study were compared with those previously found using Fisher’s exact test comparing two polymorphism groups (2 + 3 *versus* 3a + 4 + 5) from our study *versus* those in European/Middle East studies: Deckert [24] and Yermiya [25]. From our study, frequencies of genotypes/and alleles in patients with ADS and control groups were compared using the Pearson’s chi-square tests (IBM SPSS Statistics 20; IBM. Inc.: StatSoft, Poland; *p*-values not given) and exact tests: including Fisher’s, Z-pooled, Z-unpooled, Boschloo, and Santner and Snell exact tests (R package Exact, [26]; Fisher’s test *p*-values given in Table 1, Table 2, Table 3, Table 4 and Table 5. Fisher power studies were conducted according to Lesch typology, and effect sizes detectable (for the sample sizes in this study) given in the Tables correspond to power >80%. 

## 3. Results

Allele/genotype frequencies in our study were found to be comparable with those from previous studies: Fisher’s exact test *p*-values: 0.763, 0.178 and 0.393 for comparisons between our study and previous study male, female and combined polymorphism groups. Table 1 to Table 5 present an association analysis with regard to genotypes/alleles. No statistically significant differences were observed between AD and control groups, either within the entire group or specific subgroups according to Lesch typology, using Fisher’s exact tests. Additionally, Z-pooled, Z-unpooled, Boschloo, and Santner and Snell exact tests, which often have greater power than Fisher’s test, and no statistically significant differences were observed for either group.

**Table 1:** Frequency of genotypes of the polymorphism *uVNTR* of the *MAOA* gene in patients with alcohol dependence (AD) and in controls.

Group	n	MAO A VNTR		p/Effect Size Detectable (%) **
		4 Repeat n (%)	3 Repeat n (%)	
AD patients	370	255 (0.69)	115 (0.31)	0.618/0.14
Control group *	168	112 (0.67)	56 (0.33)	

* Allele frequencies similar to previous studies as determined by Fisher tests. ** Effect size detectable using Fisher’s exact with power >80%.

**Table 2:** Frequency of genotypes of the polymorphism *uVNTR* of the *MAOA* gene in patients with alcohol dependence (AD) with Lesch type I and in controls.

Group	n	MAO A VNTR		p/Effect Size Detectable (%) **
		4 Repeat n (%)	3 Repeat n (%)	
AD—type I	105	71 (0.67)	34 (0.33)	0.895/0.18
Control group *	168	112 (0.67)	56 (0.33)	

* Allele frequencies similar to previous studies as determined by Fisher tests. ** Effect size detectable using Fisher’s exact with power >80%.

**Table 3:** Frequency of genotypes of the polymorphism *uVNTR* of the *MAOA* gene in patients with alcohol dependence (AD) with Lesch type II and in controls.

Group	n	MAO A VNTR		p/Effect Size Detectable (%) **
		4 Repeat n (%)	3 Repeat n (%)	
AD—type II	86	62 (0.71)	24 (0.29)	0.396/0.20
Control group *	168	112 (0.67)	56 (0.33)	

* Allele frequencies similar to previous studies as determined by Fisher tests. ** Effect size detectable using Fisher’s exact with power >80%.

**Table 4:** Frequency of genotypes of the polymorphism *uVNTR* of the *MAOA* gene in patients with alcohol dependence (AD) with Lesch type III and in controls.

Group	n	MAO A VNTR		p/Effect Size Detectable (%) **
		4 Repeat n (%)	3 Repeat n (%)	
AD—type III	92	59 (0.64)	33 (0.36)	0.684/0.20
Control group *	168	112 (0.67)	56 (0.33)	

* Allele frequencies similar to previous studies as determined by Fisher tests. ** Effect size detectable using Fisher’s exact with power >80%.

**Table 5:** Frequency of genotypes of the polymorphism *uVNTR* of the *MAOA* gene in patients with alcohol dependence (AD) with Lesch type IV and in controls.

Group	n	MAO A VNTR		p/Effect Size Detectable (%) **
		4 Repeat n (%)	3 Repeat n (%)	
AD—type IV	87	63 (0.72)	24 (0.28)	0.394/0.20
Control group *	168	112 (0.67)	56 (0.33)	

* Allele frequencies similar to previous studies as determined by Fisher tests. ** Effect size detectable using Fisher’s exact with power >80%.

## 4. Discussion

The results of several recent studies on the association between alcohol dependence and the MAOA polymorphism, are conflicting [27,28,29]. Decreased platelet MAO activity has the characteristics of a “trait marker” in alcohol-dependent subjects compared with controls, as it persists in periods of abstinence from alcohol [30,31]. Lower platelet MAO activity was found to be lower in AD subjects with affected relatives [30,31,32]. This has strengthened the concept that MAO is a marker of genetic susceptibility. Low MAO is claimed to be a feature of “male” type 2 AD in Cloninger typology [1,27,33,34,35,36], despite the fact that low activity has also been reported in female AD subjects [37,38]. Cloninger type 2 AD includes early onset, poor impulse control and social problems such as violence while intoxicated [35,36] According to [39] *MAOA* gene might be related to antisocial AD, at least in Caucasian males. Exons 8 and 14 of the *MAOA* gene were screened and association analysis was performed for both individual polymorphisms and haplotypes. Significant differences were found between these two groups. The functional low-activity 3-repeat allele of the *MAOA* promoter polymorphism is associated with antisocial AD in German males [9,40]. Functional polymorphisms in the *MAOA* gene would be good candidate variants for associations with susceptibility to AD. However, the association between the *MAOA* gene and AD with or without antisocial personality disorder (ASPD) is not universally acknowledged. Few studies have found no association between the *MAOA* gene and antisocial AD in Caucasians, Finns, and Han Chinese [11,41,42].

Abnormal aggressive behavior in the male members of a Dutch family with a complete MAOA deficiency caused by a point mutation in exon 8 of *MAOA* gene was reported. The finding suggested a link between aggressive behavior and MAOA activity [43]. Males severely maltreated as children and having low-activity *MAOA*-*uVNTR* 3-repeat polymorphism were more likely than controls to develop antisocial behaviors as adults [44]. A possible relationship between *MAOA* gene and antisocial AD in Caucasian males was reported by [39]. A lack of association between antisocial ALC and the *MAOA* gene in the Han Chinese population in Taiwan were established [11]. Dopamine is metabolized to DOPAL by MAOA [45,46,47]. When metabolite pathways are influenced by the high-activity of the *MAOA* 4-repeat allele, the dopamine level might decrease.

The results presented in our paper include no statistical significance Table 1, Table 2, Table 3, Table 4 and Table 5. The analysis was performed with regard to the genotypes within the specific subgroups of alcoholics according to Lesch typology. A genetic variation in a variable nucleotide repeat (VNTR) located immediately upstream of the MAOA minimal central promoter has been already associated with different vulnerability to ASPD [13,40,48] and two forms of SUD: alcohol dependence (AD) [9,13,40,42] and nicotine dependence (ND) [49]. As a result, given the previous reports of GxE effects at this locus with respect to ASPD [44], it is reasonable to hypothesize that epigenetic processes, such as methylation, which affect MAOA activity may also be a factor with respect to these disorders at the MAOA locus [50,51].

## 5. Conclusions

Lesch typology does not include patients with antisocial personality. That could be a possible reason that we failed to find associations between the subgroups of patients and this polymorphism. Another limitation of the study was that the minimum effect size difference detectable (using Fisher’s tests) was 14%, and therefore we could not exclude possible differences below this detection limit.

Remarkably, the low-activity allele of the MAOA-VNTR polymorphism carriers in the literature are significantly more often present in patients with antisocial personality and in those with higher aggression. The Lesch typology of alcoholism does not specify such a specific group of alcoholics, they are rather classified as type IV with “organic disturbances”. That may be one of the reasons why we failed to find associations with this polymorphism using Lesch typology.
