# Correction: Samochowiec, A., *et al.* Monoamine Oxidase A Promoter Variable Number of Tandem Repeats (*MAOA*-*uVNTR*) in Alcoholics According to Lesch Typology. *Int. J. Environ. Res. Public Health* 2015, *12*, 3317–3326

**DOI:** 10.3390/ijerph120606892

**Published:** 2015-06-16

**Authors:** Agnieszka Samochowiec, Magdalena Chęć, Edyta Kopaczewska, Jerzy Samochowiec, Otto Lesch, Elżbieta Grochans, Andrzej Jasiewicz, Przemyslaw Bienkowski, Łukasz Kołodziej, Anna Grzywacz

**Affiliations:** 1 Institute of Psychology, Department of Clinical Psychology, University of Szczecin, Krakowska 69, 71-017 Szczecin, Poland; E-Mails: samoaga@tlen.pl (A.S.); magda.chec@gmail.com (M.C.); 2 University Center for Education, University of Szczecin, Szwoleżerów 18a, 71-062 Szczecin, Poland; E-Mail: edi55@wp.pl; 3 Department of Psychiatry, Pomeranian Medical University, Broniewskiego 26, 71-460 Szczecin, Poland; E-Mails: samoj@pum.edu.pl (J.S.); andrzej.jasiewicz@gmail.com (A.J.); 4 Department of Psychiatry, Vienna University, Waehringer Guertel 18–20, A-1090 Vienna, Austria; E-Mail: otto-michael.lesch@meduniwien.ac.at; 5 Department of Nursing, Pomeranian Medical University, Żołnierska 48, 71-210 Szczecin, Poland; E-Mail: grochans@pum.edu.pl; 6 Institute of Psychiatry and Neurology, Department of Pharmacology, Sobieskiego 9, 02-957 Warszawa, Poland; E-Mail: bienkow@ipin.edu.pl; 7 Department of Orthopedics, Pomeranian Medical University, Unii Lubelskiej 1, 71-460 Szczecin, Poland; E-Mail: Lukas@hot.pl

The authors wish to make the following corrections to this paper [1]: 

The author name “Kołodziej Łukasz” should be “Łukasz Kołodziej”.

The authors would like to apologize for any inconvenience caused to the readers by these changes.
